# Using contrast-enhanced ultrasonography to assess the degree of acute testicular torsion: a case series

**DOI:** 10.1186/s12880-022-00953-9

**Published:** 2022-12-17

**Authors:** Bin Zou, Fuqiang Zeng, Yuling Yang

**Affiliations:** 1 Department of Ultrasound, Zhongshan Hospital of Traditional Chinese Medicine, 528400 Guangdong, China; 2 Department of Medical Imaging, Zhongshan Hospital of Traditional Chinese Medicine, 528400 Guangdong, China

**Keywords:** Testicular torsion, Ultrasonography, Contrast enhancement, Doppler

## Abstract

**Background:**

There are two types of testicular torsion: complete and incomplete. The degree and duration of symptoms of this condition are critical for treatment decision-making, as the consequences for untimely diagnosis and management can be serious. The preoperative assessment of the degree of acute testicular torsion using ultrasonography is particularly important for determining the appropriate intervention. The purpose of this study was to compare the effectiveness of high-frequency versus contrast-enhanced ultrasonography in determining the degree of acute testicular torsion.

**Methods:**

Fifteen patients with clinically diagnosed acute testicular torsion underwent both high-frequency and contrast-enhanced ultrasonography. We compared the characteristics of the ultrasonographic images of the testicular parenchyma in both the afflicted and contralateral (healthy) testes to determine the reliability of contrast-enhanced ultrasonography in assessing the degree of acute testicular torsion.

**Results:**

The high-frequency ultrasound and contrast-enhanced ultrasound diagnosis of 4 complete testicular torsion and 11 incomplete testicular torsion were correct before operation. However, 5 patients with incomplete testicular torsion were misdiagnosed as complete testicular torsion because no blood flow was detected by high frequency ultrasound. Finally, low speed blood flow was detected by contrast-enhanced ultrasound and the diagnosis was corrected. The accuracy of diagnosing incomplete testicular torsion was 100% using contrast-enhanced ultrasonography and 66.7% using high-frequency ultrasonography; the difference between the two methods was statistically significant (χ^2^ = 2.50, *P* ≤ 0.05).

**Conclusion:**

Contrast-enhanced ultrasonography can diagnose testicular torsion with high accuracy and can detect low-velocity blood flow and show microcirculatory blood perfusion in the testicular parenchyma. This can avoid misdiagnosing incomplete testicular torsion as complete, thus averting unnecessary orchiectomy.

## Background

Testicular torsion is a common cause of acute scrotal distension, which occurs especially in children and adolescents but can occur in adults; it has an incidence rate of 1/4000 [1]. Awareness of the degree and timing of torsion is critical for determining the appropriate treatment and for avoiding serious repercussions arising from untimely diagnosis and incorrect management. Complete testicular torsion is usually treated by surgical removal of the affected testicle, as the lack of blood supply and perfusion precludes the restoration of testicular function [2]. However, in patients with incomplete testicular torsion, the blood supply to the testis can be significantly improved or even restored through its manipulation or surgical preservation, given that blood perfusion is still present. Testicular torsion occurs most commonly among children, for whom removal of the testis can cause permanent physical and emotional harm; therefore, the key to improving outcomes and reducing adverse consequences lies in the early, rapid, and accurate diagnosis of this condition [3, 4]. The differential diagnosis of complete versus incomplete testicular torsion is heavily dependent on the detection of subtle blood flow in the torsioned testis; this can help determine whether the organ can be preserved by restoring blood reperfusion, which would avert negative sequelae in terms of the patient’s quality of life. High-frequency ultrasonography (color Doppler) is generally the first-choice modality for the diagnosis and evaluation of testicular torsion [5]; however, this method is not sensitive to low-velocity testicular blood flow. Meanwhile, contrast-enhanced ultrasonography has the unique advantage of being able to visualize microcirculatory perfusion in the testicular parenchyma and detect very low-velocity blood flow in microcirculatory fine vessels. The purpose of this study was to compare the effectiveness of high-frequency versus contrast-enhanced ultrasonography in determining the degree of acute testicular torsion.

## Methods

### Patients

The inclusion criteria of this study were patients with incomplete testicular torsion confirmed by surgery. The exclusion criteria consisted of the following : (1) patients with complete testicular torsion; (2) patients without surgical confirmation and conservative treatment; and (3) patients who cannot cooperate with ultrasonic examination before and after treatment. All patients were admitted to the emergency department of Zhongshan Hospital of Traditional Chinese Medicine between January 2021 and September 2022 and suspected of testicular torsion after preliminary diagnosis by clinicians. A total of 15 patients with acute incomplete testicular torsion (all with unilateral onset; 6 on the right side and 9 on the left side) were enrolled. They were aged 9–74 years; 9 were children or adolescents and 6 were adults. The patients presented with a sudden onset of scrotal swelling and pain, and all underwent high-frequency as well as contrast-enhanced ultrasonography. The study was approved by the Ethics Committee of Zhongshan Hospital of Traditional Chinese Medicine. All methods were performed in accordance with relevant guidelines and regulations. Each of the subjects and/or their representatives was instructed on the purpose of the study and signed an informed consent form.

### Ultrasonographic examination

The Hitachi Spiral 850 Ultrasound Diagnostic Instrument, Toshiba Aplio 500 Ultrasound Diagnostic Instrument, and GE-logicE9 (probe frequency 10–14 MHz, equipped with the ‘superb microvascular imaging’ function) were used for the examinations. Each patient was instructed to lie in a supine position and fully expose the scrotum. The positions of both testes as well as their morphologies, sizes, envelope integrity, and internal echogenicity were observed with high-frequency ultrasonography using the 2-dimensional mode. Subsequently, the sampling frame was adjusted to the low-speed scale, and multiple sections of both testes were scanned using high-frequency color ultrasonography without changing the machine’s settings to observe the blood flow. The images were stored for graphical analysis, and testicular torsion was diagnosed based on the obtained 2-dimensional and color images. Next, we used contrast-enhanced ultrasonography that was tuned to low mechanical index imaging; 2.4 ml SonoVue contrast agent was injected through the left or right median vein by the same ultrasound doctor who observed testicular parenchymal blood perfusion from multiple angles and sections in real time without interruption for 3 min, focusing on the testicular blood perfusion characteristics, distribution, and contrast filling of any defects on both sides.

### Statistical analysis

The data were analyzed using SPSS statistical software version 25.0 (IBM Corp., Armonk, NY, USA). The diagnostic accuracy of high-frequency and contrast-enhanced ultrasonography was compared using the χ^2^ test in a paired design, with differences considered statistically significant at a *P*-value ≤ 0.05.

## Results

### Patients

Nine of the 15 patients were found to have complete testicular torsion using high-frequency ultrasonography; conversely, contrast-enhanced ultrasonography indicated that only 4 of them had complete torsion, while the remaining 9 had incomplete torsion. Furthermore, the remaining 6 patients were found to have incomplete torsion using both modalities. The interval between symptom onset and diagnosis of testicular torsion ranged from 1 to 6 h, with a mean of 3.4 h. Subsequently, the patients’ diagnoses were confirmed by surgery, which revealed that 4 and 11 had complete and incomplete testicular torsion, respectively; this was consistent with the contrast-enhanced ultrasonography findings.

### Assessment of the torsioned testicle

Four patients with complete testicular torsion as confirmed surgically showed morphological derangement, enlargement, and hypoechogenicity of the affected testis with no detectable blood flow on high-frequency ultrasonography. No contrast accumulation was observed inside the testicular parenchyma on contrast-enhanced ultrasonography.

**Fig. 1:**
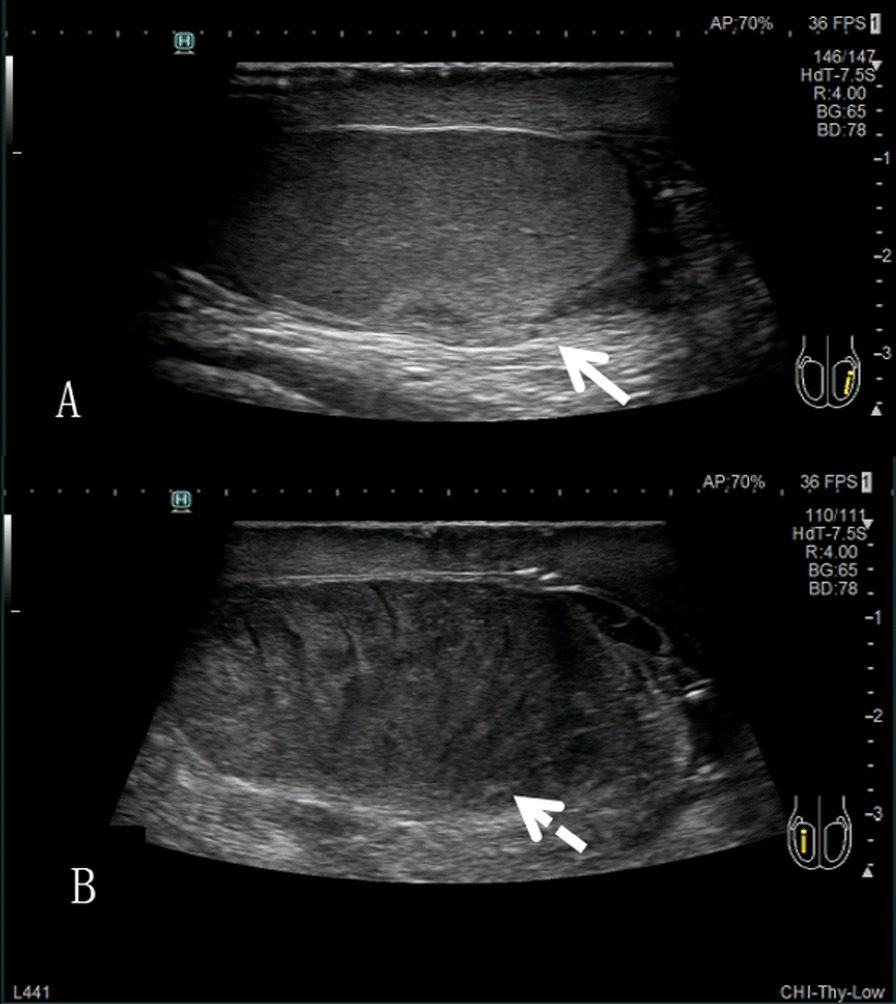
Normal testis (**A**) and abnormal testicular (**B**) echotexture. Patient 1. Male, 19 years old. The patient with incomplete torsion, high-frequency ultrasonography showed normal echo of healthy testis (**A**), decreased echo of affected testis (**B**)

**Fig. 2:**
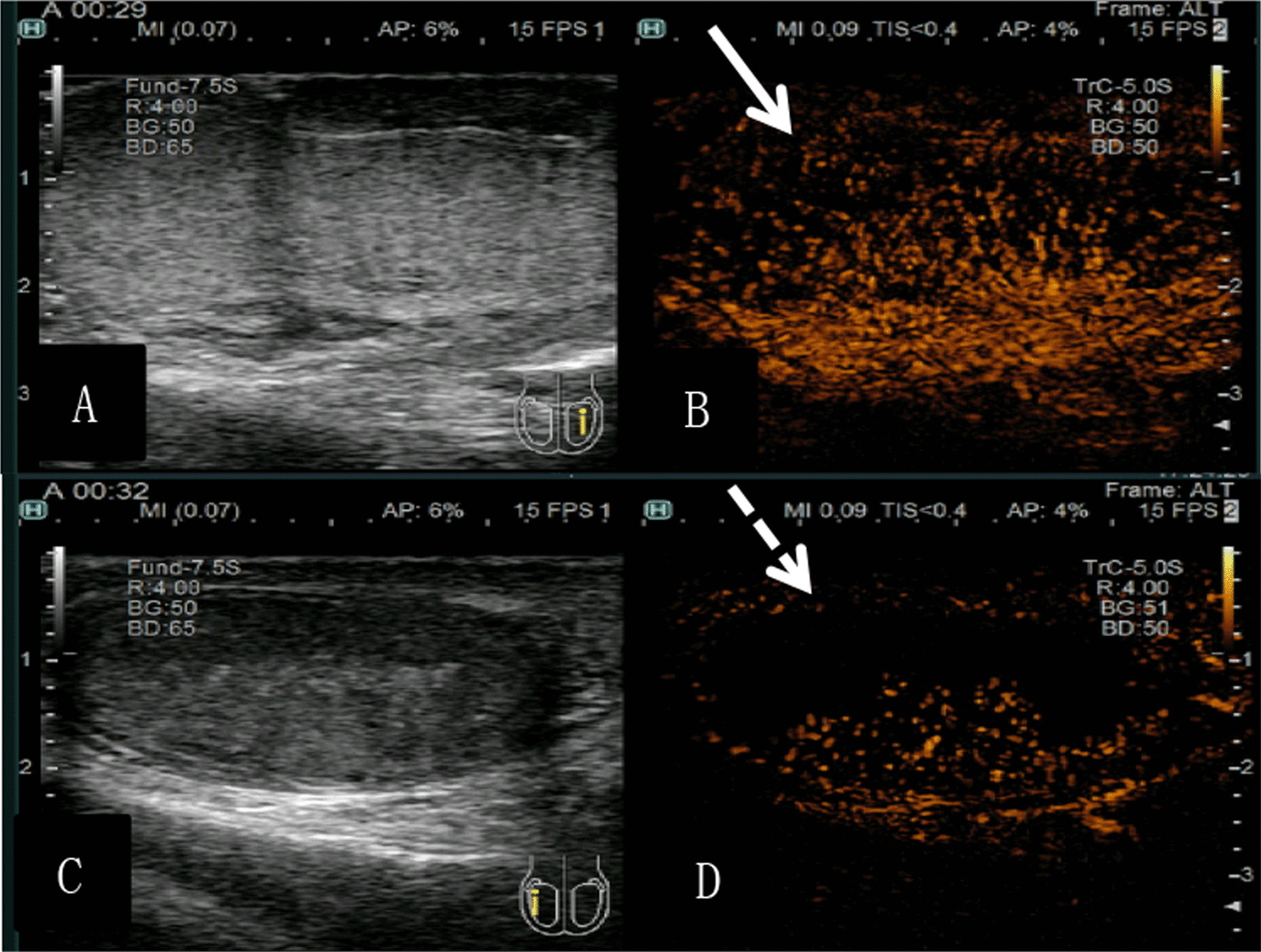
Normal testis using high-frequency ultrasound (**A**) and CEUS (**B**), testicular torsion using high-frequency ultrasound (**C**) and CEUS (**D**). Patient 2. Male, 19 years old. The patient with incomplete torsion. High-frequency ultrasonography showed normal echo of healthy testis (**A**), contrast-enhanced ultrasound showed that the this normal testicular parenchyma is filled with contrast agent with evenly distributed and highly enhanced (**B**); high-frequency ultrasonography showed abnormal echo of healthy testis (**C**), contrast-enhanced ultrasound showed that the this twisted testicular parenchyma was filled with contrast agents with uneven distribution, and the enhancement degree was lower (**D**).

Among 11 patients ultimately diagnosed with incomplete testicular torsion, high-frequency ultrasonography showed no significant changes in the echogenicity of the affected testis in 5 of them. Moreover, the morphology of the affected testis in these patients was altered, and there was no detectable blood flow in any of them. Therefore, these patients were misdiagnosed by high-frequency ultrasound. In contrast, a trace blood flow signal was detected in all 5 when using contrast-enhanced ultrasonography. Finally, these patients were correctly diagnosed by contrast-enhanced ultrasound before surgery. In the remaining 6 patients with incomplete torsion, high-frequency ultrasonography showed reduced testicular echogenicity compared to the contralateral side (Fig. 1), and trace blood flow could be detected. Contrast-enhanced ultrasonography revealed that the testicular parenchyma was filled with unevenly distributed contrast agent, with the degree of enhancement lower than that on the healthy side (Fig. 2). Therefore, these patients were correctly diagnosed by both high-frequency and contrast-enhanced ultrasound before surgery.

**Fig. 3:**
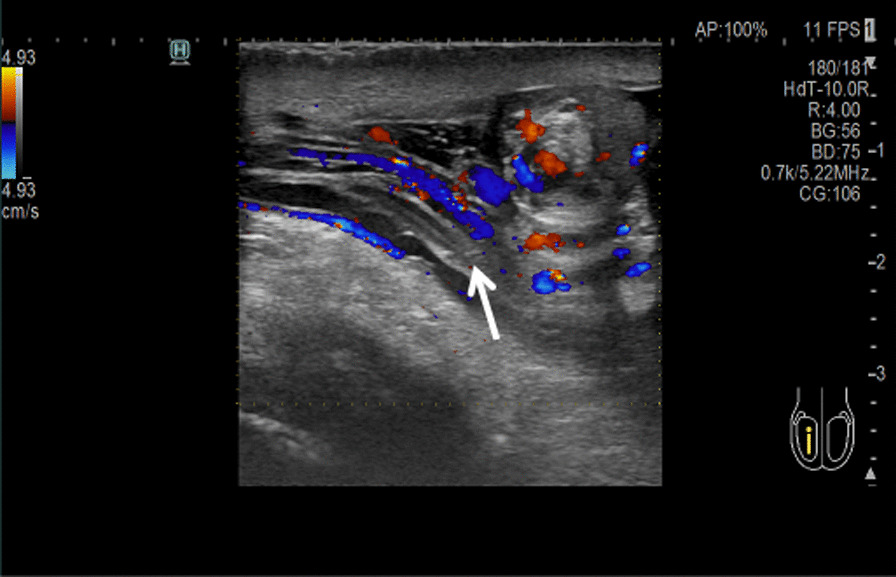
Spiral torsion of spermatic cord above testis. Patient 3. Male, 14 years old. The torsioned spermatic cord exhibited echogenic enhancement and was rich in blood flow

Scrotal fluid accumulation was observed in 9 of the patients with incomplete torsion, whereas 2 experienced an increase in epididymal volume. In all 11 of these patients, spiral torsion of the spermatic cord was observed above the testis. The torsioned spermatic cord exhibited echogenic enhancement and was rich in blood flow (Fig. 3); the mass rotated when the probe was moved up and down.

The accuracy of high-frequency ultrasonography in diagnosing the degree of testicular torsion was 66.7% (10/15), which was lower than that of contrast-enhanced ultrasonography (100%, 15/15). The difference in diagnostic accuracy between the two methods was statistically significant (*P* ≤ 0.05). The patients’ data are summarized in Table 1.

**Table 1:** Summary of ultrasonographic findings in 15 patients with surgically confirmed testicular torsion

Surgical findings	Number of patients	Changes in testicular echogenicity	Morphological changes	High-frequency ultrasonography blood flow signal	Ultrasonography with intratesticular contrast	Scrotal effusion	Enlarged epididymis
Complete testicular torsion	4	4	4	None	None	4	2
Incomplete testicular torsion	11	5	6	None in 6 Trace signal in 3	Trace signal in 3	9	2

## Discussion

The degree and duration of testicular torsion are closely related to the treatment method: the more extensive the torsion and/or the longer the torsion time, the more severe the ischemia and the lower the probability of preserving the testis. Clinical methods for diagnosing testicular torsion usually involve ultrasonography; high-frequency color ultrasonography is widely used for its sensitivity and accuracy in diagnosing complete testicular torsion. However, owing to the physical characteristics of ultrasound itself, color Doppler is not sensitive to low-velocity blood flow. In the early stages of incomplete testicular torsion, the spermatic vein is compressed and blood return in the testis is impaired; however, the testicular artery can still deliver blood [6], and the testis can appear relatively normal on high-frequency ultrasonography. Moreover, there may be no significant changes in testicular blood perfusion when examined with the naked eye [7], and some incomplete torsions may be missed [8]. As the course of testicular torsion progresses, the damage to the testis increases while the angle of spermatic cord torsion becomes too large; this compresses the testicular artery, allowing only a small amount of low-velocity arterial blood to flow. This in turn may be misdiagnosed as complete testicular torsion when using single high-frequency color ultrasonography [9]. As such, this modality has limited applicability in terms of correctly diagnosing incomplete testicular torsion [10]. Additionally, patients suspected of having acute testicular torsion are usually children who tend to have poor cooperation during examinations, resulting in incorrect blood flow assessment that can lead to misdiagnosis. Some patients also have poorly-developed testes with slender internal blood vessels and sparse or slow blood flow, making it more difficult to visualize the blood flow signal on high-frequency color ultrasonography [11].

Conversely, CEUS has a good application in non-invasive evaluation of blood perfusion of tumors and target organs, and its value is reflected in the European clinical contrast-enhanced ultrasound guidelines developed by the European Commission for Ultrasound Medicine and Biomedical Sciences (EFSUMB) [12, 13]. Ultrasound contrast agent (SonoVue) is a blood pool tracer, which can overcome the physical limitations of high-frequency color ultrasound and make up for the lack of high-frequency color ultrasound in monitoring the low-speed blood flow in small vessels [14–17]. Therefore, we believe that contrast-enhanced ultrasonography also dynamically displays the perfusion characteristics of the contrast organ and microvascular imaging characteristics and accurately reflects the hemodynamic changes of the microcirculation, thus improving the display rate of microcirculatory vessels and avoiding the misdiagnosis of incomplete testicular torsion as complete. Therefore, this prevents adverse consequences, such as testicular resection. In some patients with minor incomplete testicular torsion, the changes in blood flow characteristics are so small that they cannot be perceived by the naked eye and are easily missed. In these cases, quantitative ultrasonographic analysis can accurately detect the subtle differences in contrast perfusion between healthy and torsioned testes, which—when combined with the patient's clinical symptoms—provides the basis for the accurate preoperative diagnosis of incomplete testicular torsion.

In this study, 11 patients with incomplete testicular torsion saw uneven distribution of contrast agent filling inside the testes, and 5 patients were misdiagnosed as complete testicular torsion by high-frequency color ultrasound. However, a small amount of contrast agent filling was still seen inside the testes, but the echo intensity was significantly lower than that of the healthy side, showing extremely low enhancement. Furthermore, the local defect area of contrast agent was visible, which was consistent with the results of surgical verification. This shows that high-frequency ultrasound can only show the shape, size, signal and blood supply of the testicular parenchyma, while contrast-enhanced ultrasound can monitor the low-speed blood flow in microvessels through the injection of contrast agents and can more accurately find subtle and low-speed blood flow signals. In addition, the main component of the ultrasound contrast agent is sulfur hexafluoride microbubbles, which are discharged from the body through respiratory metabolism.However, toxicity from the injection of contrast-enhanced ultrasound may cause mild adverse reactions, such as headache, nausea, and sweating. In orderfor patients to recover in a short time and with no residual effects, they need to be properly informed and sign the consent form before examination. Our data show that contrast-enhanced ultrasound has a unique advantage in evaluating microcirculation perfusion of testicular parenchyma, so as to detect very low velocity blood flow in microcirculation small vessels. This makes it an ideal choice to evaluate the degree of acute testicular torsion and observe the ischemia-reperfusion after testicular surgery. However, because the sample size of this study is not large enough, the quantitative standard still needs to be further verified by collecting more cases in future studies, which is the next research direction of the author.

## Conclusion

We found that contrast-enhanced ultrasonography can accurately visualize the microcirculatory perfusion characteristics of the testicular parenchyma, and that it can diagnose incomplete testicular torsion more accurately than high-frequency ultrasonography. As such, contrast-enhanced ultrasonography can provide clinicians with accurate quantitative information when evaluating patients with suspected testicular torsion, compensates for high-frequency ultrasonography’s deficiency regarding microvessels and low-speed blood flow, and promotes the application of ultrasound technology in the preoperative evaluation of testicular torsion, and thus aids in devising treatment options.

## Data Availability

The authors declare that all data collected during this study can be found in the paper, while the source data for the figures and tables in the study are available from the corresponding authors upon request.
